# Hidden in the photograph: The myth of complete metabolic coverage possible in metabolomics investigations

**DOI:** 10.1002/ansa.202200055

**Published:** 2023-03-21

**Authors:** C. Benjamin Naman, Sajeevan Thavarool Puthiyedathu, ChaeYeon C. Poulin, Remington X. Poulin

**Affiliations:** ^1^ Department of Science and Conservation San Diego Botanic Garden Encinitas California USA; ^2^ National Centre for Aquatic Animal Health Cochin University of Science and Technology Kochi India; ^3^ Department of Chemistry and Biochemistry Center for Marine Science, College of Arts and Sciences University of North Carolina Wilmington Wilmington North Carolina USA

## Abstract

Since the late 1970s, many ‘omics‐style investigations have advanced our understanding of systems at all levels, from community level, through organismal, to individual cellular processes. Beginning with genomics and progressing through transcriptomics, proteomics and finally to metabolomics, the scope of interest shifts significantly from what is genetically possible to what is currently expressed, produced and measurable in a system. While the ideal goal of any ‘omics investigation is to fully describe a system, loss of information occurs at each decision‐making juncture. These losses are often not considered in the experimental planning stage but, when combined, they drastically affect the power of an investigation and the conclusions that can be drawn from it. Herein we discuss through the analogy of photography many of the decision‐making junctures of metabolomics investigations and the resultant losses of information occurring at each.

## 1 INTRODUCTION

In the ever‐waging race of instrumental advances, software development and big data science, ‘omics investigations are becoming increasingly more common. ‘Omics‐style investigations, which include genomics, transcriptomics, proteomics and metabolomics, combine the detectable DNA, mRNA, protein and metabolite data to provide a biochemical snapshot of a system (e.g. cell, tissue, organism, population, community, etc.) (See Hasin, Seldin and Lusis for a generalized overview of ‘omics and their integration).^1^ The more complete the description of the system, the more thoroughly it can be investigated for the factors that affect the system and their resultant physiological, chemical and behavioural responses.

**FIGURE 1:**
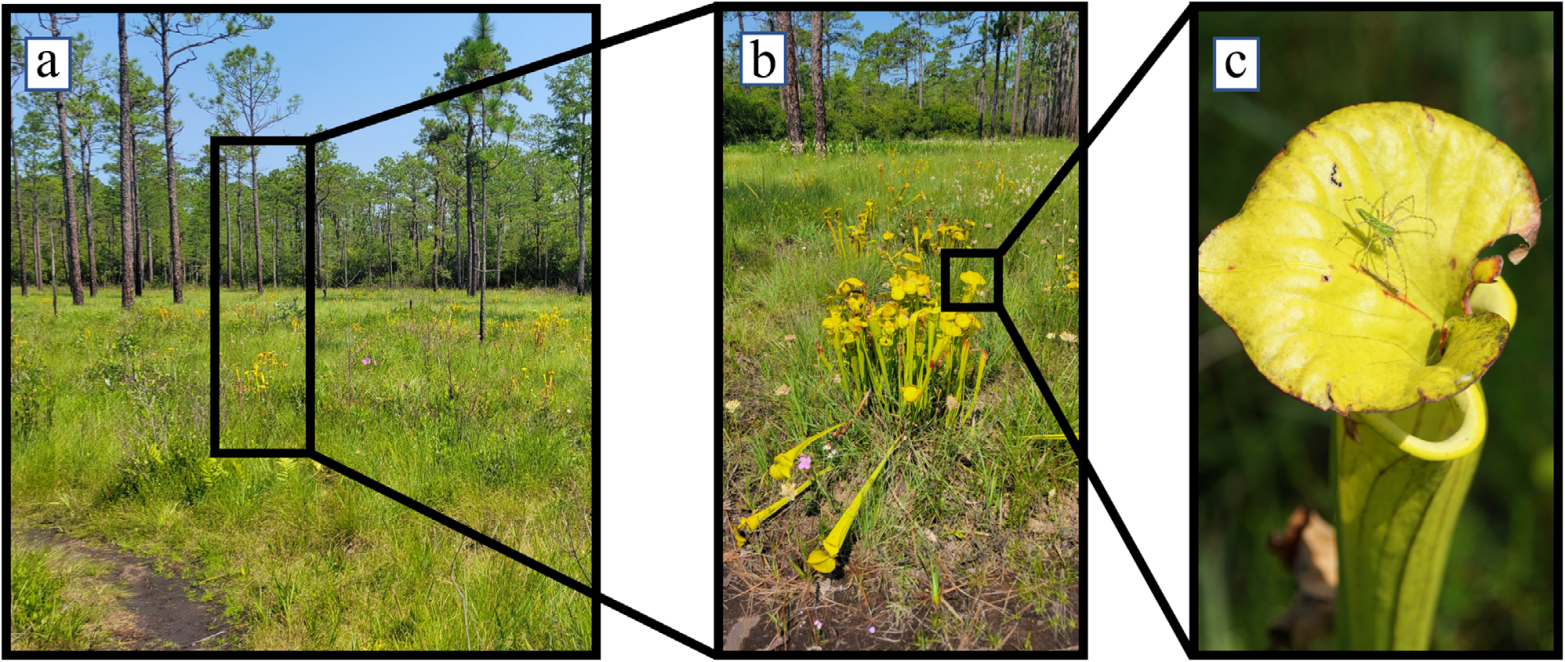
Photographic series of a swamp highlights the amount of information gained and lost, by framing and selecting a subject of interest. (A) An image of a long leaf pine swamp in Southeastern North Carolina. (B) a zoomed‐in image showing more details of a patch of endangered pitcher plants. (C) a further magnified image of a single pitcher plant showing a camouflaged green spider only visible with significant focus and zoom. As incremental zoom is applied, left to right, a–c, information is gained on the plants and eventually spider but information on the color of the sky, the height of the trees, the identities of surrounding foliage and more are lost simultaneously.

Many studies draw the analogy of a photograph to that of an ‘omics investigation—what can be seen in the photograph represents the current understanding of the system as it can be measured. In a photo of a swamp, for example (Figure 1A), the diversity of vegetation, the blue colour of the sky, the size of the path in the bottom left and much more are observable. If the photo is zoomed on a clump of endangered pitcher plants, additional insight into the number of individual pitchers, how they arrange within the clump and their colourations and markings are gained (Figure 1B). However, this is done at the cost of losing information on the dispersal of pitcher plant clumps in the swamp and how tall the trees are. If the focus is even further placed on an individual pitcher (Figure 1C), a green spider camouflaging with the green veins of the plant can be observed. This zoom and focus, while making the spider visible, again leads to a significant loss of information about the overall swamp. The camera, lens, settings and other factors also had to be adequately selected to produce the photo quality and focal point of recorded information as presented. It is clear then that as decisions are made on the subject matter, understanding of the system is compromised as tradeoffs between breadth and specificity are navigated.

This analogy perfectly mimics that of metabolomics investigations—as studies proceed from sample generation through data collection ending in data processing/analysis, the view of the swamp for the spider or system for the metabolite can be lost. While each of the individual ‘omics investigations has its own considerations, herein we provide a systematic overview of the many obstacles that prevent the collection of a complete metabolomic profile of a system.

## 2 TAKING THE SNAPSHOT

### 2.1 Focusing on subject matter versus biological sample choice

**FIGURE 2:**
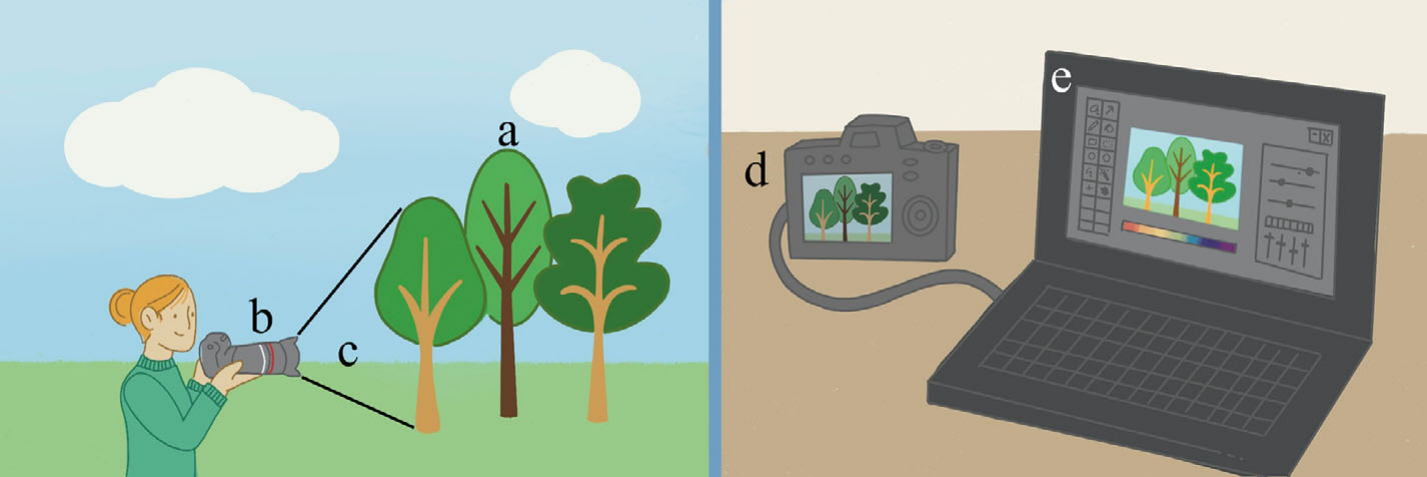
Metabolomics investigations are analogous to photography. (A) Subject matter of a photo is identified and focused on—biomass of interest is collected. (B) exposure and camera settings, or light filtering lenses, are selected that best fit the subject matter—metabolism is quenched and extracted. c) subject matter is framed via adjusting zoom or location of the photographer—extracts are processed. d) capture and digitization of images via camera computer—extracts are profiled via analytical instrumentation. e) images are processed—metabolic profiles are analyzed and individual metabolites are identified via databases.

The most vital aspect of a photograph or metabolomic snapshot is, of course, the subject matter (Figure 2A). A photographer does not attend a sporting event to take nature photographs and consequently, a leaf is not collected if root biochemistry is of interest. To photograph any scene in sufficient detail, an appropriate focus on the correct subject matter is required. Significant effort must likewise be made to ensure that enough metabolomics material, or biomass, is available and that this corresponds to the level of detail of the planned experiments (more biomass leads to higher quantities of all and thus the less abundant, metabolites). The first obstacle in collecting a complete metabolome is the insufficient availability of material, which will lead to a bias towards only the most abundant metabolites. In many systems, vital metabolites, such as chemical defences, toxins, pheromones, etc. are produced in incredibly low concentrations but elicit potent responses. This obstacle however is mitigated in part by instrumental advances that allow for faster sampling rates, higher sensitivity and overall enhanced data quality, which is discussed more thoroughly in section 4.

### 2.2 Exposure versus metabolic quenching and extraction

As many good photographers know, manipulating the exposure time of a photograph drastically affects the resultant picture. The longer the lens is left open, the more light reaches the camera sensor and more information is collected. Long‐exposure photography can lead to blurred images of moving objects such as waterfalls and cars, or it can be used to collect stunning nighttime photographs where long exposure is required to collect enough light. Conversely, very short photographs can lead to crisp images of fast‐moving objects in high‐light environments, as would be desired at sporting events. Physical lens filters can also be applied, for example, to include or exclude light via polarization or wavelength. Thus, appropriate prior decisions must be made because over or underexposure can dramatically decrease the overall goodness of the photograph by obscuring desired details.

In the same way that exposure time is important in photography (Figure 2B), quenching metabolism and extracting the resultant metabolites is important. The metabolic timescale varies depending on the specific physiological response investigated, so understanding the exact moment of metabolic quenching is vital to understanding which biochemical processes could be occurring. The most rigorous metabolomic studies aim to quench metabolism instantaneously to ensure that the specific metabolites observed are due to host metabolic activity and not from other processes post biomass collection. This can be conducted using numerous methodologies, most of which employ reduced temperatures.

Once the metabolism is quenched, extracting metabolites from biomass becomes the next hurdle. Extraction of metabolites is most commonly completed using a mid‐polarity solvent such as acetone, mixtures of solvents that better span the polarity range (i.e. a methanol chloroform mixture), or in the most expansive investigations, conducting a series of iterative extractions of biomass through a range of solvents spanning the full polarity range. In all three cases, a set of metabolites are excluded or have their relative concentrations significantly altered due to incomplete extraction. For these reasons, the extraction stage of a metabolomics study is one of the major steps in affecting the incomplete coverage of the metabolome.

## 3 PROCESSING SNAPSHOT

The processing of a picture can produce the illusion of a different capture method. For example, applying wavelength shifts may correct for or otherwise augment the lighting, colouration, contrast and saturation of the image. So too can metabolomic sample processing augment the composition of the mixture in obvious or obscure ways. A typical first step for sample processing is the concentration of an extract since relatively large volumes of a solvent tend to be used in their preparation. Until the sample has been processed, the content remains unclear due to the complexity of the extract and the generally low concentration of analytes in the solution. The conditions of sample concentration, including temperature and reduced pressure, but also light and air exposure, each provides the opportunity for augmentation of metabolites via different degradation pathways and means of loss. Many authors omit the details of the concentration step or lack careful control of the laboratory processes that can alter the contents of the sample, much akin to the under‐ or overdeveloping of film in classical cameras or using a loss‐prone compression algorithm or reduced resolution file to save modern digital photos.

### 3.1 Framing the image versus separation of mixtures

In the same way that a photographer will frame the subject of interest via a viewfinder (Figure 2C), post‐extraction sample processing facilitates the metabolomic investigation of complex mixtures. To reduce the complexity of the mixture in an extract, scientists often desalt samples and use other forms of offline crude separation, including liquid‐liquid partitioning, solid phase extraction for non‐volatiles and solid phase microextraction for volatiles, prior to data acquisition even with in‐line separation methods described in section 4. Just as the various portions of a scene can be excluded from a photograph by changing the zoom length, ranges of polarity metabolites can be selectively separated from an extract. There is also the opportunity during these processes for sample contamination, sample loss and sample degradation to occur that would impact the contents of the mixture and the outcomes of any later analysis. Thus it is required that the type of metabolites of interest should be determined in advance and the method of their enrichment should be declared in reports, because this directly modifies the data to be collected by creating inclusion and exclusion criteria, much like framing an image. In both cases, the result is often an imperfect selection or removal that can be improved upon by post‐processing, which is addressed in section 5.

## 4 PROFILING SNAPSHOT AND DIGITIZATION

In much the same way that a photographed scene needs to be developed and processed after being recorded onto film, then digitally scanned (for classic cameras) or directly recorded into a digital format by algorithms and a processor (using digital cameras) (Figure 2D), metabolomics investigations require the conversion of an extract to a metabolic profile via analytical instrumentation such as nuclear magnetic resonance spectrometer (NMR), liquid chromatography‐mass spectrometry (LCMS) or gas chromatography‐mass spectrometry (GCMS). A key difference, for example, is that photographers have an analogue viewfinder or digital preview when taking photographs that provide direct insight into the expected contents of the profile present before the lens. In a metabolomics investigation, however, the visual connectivity between the extract in a vial and a spectrometric or spectroscopic trace is severed. This remains much akin to the historical perspective of photographers, who could not be guaranteed that the camera and film equipment was operating correctly until the timely process of developing film had been completed later on and often without the chance to re‐take the original photograph. Likewise, a mishandled or ill‐prepared metabolomics sample may not be easily replicated.

One of the most expensive and influential tools in a metabolomics laboratory is the analytical instrumentation used to translate an extract into the associated metabolic profile. This could take the form of an NMR, or a mass spectrometer coupled to an LCMS or GCMS separation component. Each instrument requires specialized expertise from the user and will heavily influence which metabolites are observable. In many cases, the sample specifications will dictate one instrument's use over another, such as the use of GCMS with a headspace analyzer for volatile chemicals, or selecting different ionization sources for LCMS or sample probes for NMR to detect specific types of metabolites. MS is most often used in metabolomics due to its inherently higher sensitivity than NMR. However, MS also yields incomplete observation of the metabolites present and requires additional steps to make quantitative measurements. Within MS, there are instrumentation‐specific considerations, such as the type of mass detector (i.e. quadrupole time of flight vs. triple quadrupole vs. matrix‐assisted laser desorption/ionization vs. Orbitrap), the resolution and sensitivity of the detector, the ionization technique (i.e. electrospray ionization vs. atmospheric pressure chemical ionization) or the ionization mode (positive vs. negative). In some cases, multiple profiling techniques are combined to enhance coverage, however, this significantly increases the time and cost for data acquisition, as well as complicates data integration and interpretation. Each decision will further reduce the observable coverage of the metabolome of a sample and, as such, this stage is among the most reductive stages of the investigation.

## 5 SNAPSHOT ANALYSIS

A photograph defined in the context of its contents (Figure 2E) is akin to a metabolomic data set with annotations. To that end, searching a database for identical or similar entries is an excellent way to generate testable hypotheses or tentative identifications. While many reports have represented putatively annotated compounds as having been identified, the metabolomics standards initiative offers clear guidance for more accurate and appropriate nomenclature for four different levels of metabolite identification rigour based on the use of (1) orthogonal diagnostic analyses and authentic standards in the study, (2) spectroscopic and spectrometric library matching, (3) similarity to annotated molecules and (4) identification uncertainty.^2^ The same categories would hold true for photo evaluation; a single library search could yield nothing (4), a similar image (3), the same image without certainty of its authenticity (e.g. a picture of a picture, an edited picture or even the same picture saved in a different format) (2), or the original picture itself (1). Since an image search may not be able to distinguish between original photos, mirror images, reprints and other forms of copies or pictures with minor differences, these should be detected using orthogonal methods including forensic analysis, microscopic examination or even provenance studies. The metabolomic parallels include, for example, novel chemistry, configurational isomers, previously reported metabolites, constitutional isomers and other structural analogues. Clearly, only some of these can be determined by each analytical method, again highlighting the need for incorporating orthogonal approaches. Unfortunately, surprisingly few metabolomics investigations have been compliant with the minimum reporting standards, leading to undesired scepticism of many resulting claims about metabolite identifications as well as concerns over study reproducibility.^3^, ^4^

The method by which to search available databases for a picture is paramount to the outcome. One could envision searching an image bank by user‐generated keywords (such as Google image search), keywords selected from a curated dictionary (such as Google image search with annotation filters), using a full raw image (such as Google Lens), a diagnostic subsection of the image (such as Google Lens with search image cropping), or a number of other conceivable options. Searching metabolomic data is no different and removing the human element can certainly eliminate some biases and reduce error rates, for example, by using unmodified whole raw data. Furthermore, the specificity of the metabolomic information being searched is of paramount importance. In many cases, if not most, metabolites are not perfectly resolved in the separation steps jointly covered in sections 3 and 4. Thus, interpretive deconvolution of data must be conducted using the analyst's intellect and experience or preferably using computational algorithms that deconstruct complex overlapping signals and model the true contributions. A convenient example is the Automated Mass Spectral Deconvolution & Identification System software that is freely available online from the U.S. National Institute of Standards and Technology.^5^ The photography analogy of signal deconvolution would be to segregate the green spider seen in Figure 1C from the pitcher plant itself. This is something that the human mind processes during optical interpretation and analysis, but computer software and algorithms would be required if the images are to be used as library search queries separately.

Another challenge comes when choosing or even having access to an appropriate database or library to search. There is an inordinate number of databases for metabolites that can be searched in various ways, just as there are for photos and other images. Some are online and open access, others are online behind paywalls and others still are offline. Some can be searched against raw data, while others take inputs of spectroscopic or spectrometric parameters, metabolite names, structures, molecular formulae, etc. A cheminformatics review on where to find natural product data in 2020 presented the striking revelation that over 120 non‐comprehensive natural product databases existed at that time, despite many of these being relatively inaccessible.^6^ The authors thus went on to establish a new non‐comprehensive natural products database called COlleCtion of Open NatUral producTs (COCONUT) in the same year.^6^ COCONUT served to pool all of the openly available data into one library and seemingly also increased by one the total number of available natural product databases.^6^

It was not so long ago that exclusively expensive software and curated graphical collections were sold for photography editing and searching, or metabolite detection and annotation by spectrometry and spectroscopy. Meanwhile, the distribution of these has been largely reduced to specialists while free online tools have become widely normalized and yield relatively good results. Individuals are similarly left to consider the accuracy of annotations and breadth of entries in available curated, un‐curated and hybrid libraries or databases. Un‐curated data are often broadly available and can be very well or not so well annotated in the modern era, whereas vast data from decades past remains either unsearchable or unavailable without considerable financial investment to gain access to well‐curated databases. For example, the Wiley NMR library, a once‐offline and now subscription‐based curated collection of approximately one million NMR spectra, has long been considered a gold standard to those with access. However, with the goal of Findability, Accessibility, Interoperability and Reuse of data, the online and open‐access Natural Products Magnetic Resonance Database (NP‐MRD) has a few thousand experimental NMR spectra and over one million simulated and predicted NMR spectra that continue to be expanded upon.^7^ It is expected that the collective effort of dedicated individuals and forthcoming policies will contribute to the public curation of high‐quality annotations for open‐access data.

Earlier examples of openly shared libraries do exist, such as the BioMagResBank, Human Metabolome Database and Madison‐Qingdao Metabolomics Consortium Database, which can also include orthogonal data sets for metabolites.^8^, ^9^, ^10^ A dedicated set of orthogonal data to the NP‐MRD and NMR databases exists in the form of Global Natural Product Social Molecular Networking, which is an online tool for open access sharing and query of tandem mass spectrometry (MS/MS) data that has certainly expanded beyond the scope of only natural product metabolites and has furthermore become truly a global mainstay for evaluating untargeted LC‐MS/MS metabolomics data.^11^ Genomic and proteomic data has been almost globally centralized by the US National Center for Biotechnology Information, offering an exemplary means and benefit to comprehensive databasing that has not yet been matched in metabolomics. Meanwhile, a smaller but growing multi‐omics resource exists in the Kyoto Encyclopedia of Genes and Genomes, which incorporates genes, proteins and metabolites, along with pathway analysis, functional information (orthology), similarity searching and much more.^12^

With the ever‐growing quantity of online content, both photographic and ‘omics‐based, more comprehensive databases, analytical tools and computational approaches must be developed. At the same time, the amount of novel content remaining to be discovered or generated and ideally then rapidly added to databases is incrementally reduced. Thus the use of snapshot analysis tools and appropriate interpretation of search results will be increasingly informative about small and large sample sets in the context of the “big data” of all available information from prior studies, especially if comprehensive libraries can be centralized and made accessible.

## 6 CONCLUSION

No metabolomics investigation leads to total coverage. At each decision‐making juncture, some data are lost knowingly or otherwise. The keys to conducting high‐quality, reproducible metabolomics investigations are to be aware of the consequences of each methodological step, to properly and thoroughly document and disclose how samples are produced and processed and to draw conclusions that fit the remaining and removed data. Only data taken in its true context can be used to make systematic claims. To ignore everything that has been experimentally excluded is to describe a system in a proverbial vacuum that never existed; for example, the spider without a pitcher plant, a pitcher plant without its community cluster patch, or a community cluster patch without the swampy forest.

## CONFLICT OF INTEREST STATEMENT

The authors declare no conflict of interest.
